# Novel Electrospun Polylactic Acid Nanocomposite Fiber Mats with Hybrid Graphene Oxide and Nanohydroxyapatite Reinforcements Having Enhanced Biocompatibility

**DOI:** 10.3390/polym8080287

**Published:** 2016-08-08

**Authors:** Chen Liu, Hoi Man Wong, Kelvin Wai Kwok Yeung, Sie Chin Tjong

**Affiliations:** 1 Department of Physics and Materials Science, City University of Hong Kong, Tat Chee Avenue, Kowloon, Hong Kong, China; cliu266-c@my.cityu.edu.hk; 2 Department of Orthopedics and Traumatology, Li Ka Shing Faculty of Medicine, The University of Hong Kong, Hong Kong, China; kwhoiman@gmail.com (H.M.W.); wkkyeung@hku.hk (K.W.K.Y.)

**Keywords:** polylactic acid, electrospinning, graphene oxide, hydroxyapatite, nanocomposites, biocompatibility

## Abstract

Graphene oxide (GO) and a nanohydroxyapatite rod (nHA) of good biocompatibility were incorporated into polylactic acid (PLA) through electrospinning to form nanocomposite fiber scaffolds for bone tissue engineering applications. The preparation, morphological, mechanical and thermal properties, as well as biocompatibility of electrospun PLA scaffolds reinforced with GO and/or nHA were investigated. Electron microscopic examination and image analysis showed that GO and nHA nanofillers refine the diameter of electrospun PLA fibers. Differential scanning calorimetric tests showed that nHA facilitates the crystallization process of PLA, thereby acting as a nucleating site for the PLA molecules. Tensile test results indicated that the tensile strength and elastic modulus of the electrospun PLA mat can be increased by adding 15 wt % nHA. The hybrid nanocomposite scaffold with 15 wt % nHA and 1 wt % GO fillers exhibited higher tensile strength amongst the specimens investigated. Furthermore, nHA and GO nanofillers enhanced the water uptake of PLA. Cell cultivation, 3-(4,5-dimethylthiazol-2-yl)-2,5-diphenyltetrazolium bromide (MTT) and alkaline phosphatase tests demonstrated that all of the nanocomposite scaffolds exhibit higher biocompatibility than the pure PLA mat, particularly for the scaffold with 15 wt % nHA and 1 wt % GO. Therefore, the novel electrospun PLA nanocomposite scaffold with 15 wt % nHA and 1 wt % GO possessing a high tensile strength and modulus, as well as excellent cell proliferation is a potential biomaterial for bone tissue engineering applications.

## 1. Introduction

The development of polymer scaffolds with good bioactivity and biocompatibility is considered of significant technical and clinical importance due to a large increase in ageing populations, and the number of patients suffering from bone disease, trauma, traffic accident and sports activity. Nowadays, bone diseases (e.g., osteoporosis, scoliosis and tumor) and injuries cause a significant public health problem. Bone tissue generally exhibits excellent regeneration capacity and can repair itself upon injury. However, this self-healing is impaired if trauma is serious and exceeds a certain size. Tissue engineering integrates engineering and biomedical approaches to develop biocompatible scaffolds by seeding cells on their surfaces for achieving bone tissue repair and reconstruction. The aim is to restore the functions of damaged bone tissues and defects and to promote integration with the host tissue [1]. The scaffolds serve as an artificial extracellular matrix (ECM) that provides temporary structural support for cell adhesion, proliferation and bone regeneration [2,3]. In addition, the adequate mechanical strength and degradation rate of porous scaffolds are also important factors for clinical applications.

Hydroxyapatite (HA) with a chemical composition of Ca_10_(PO_4_)_6_(OH)_2_ is an ideal material for bone replacements owing to its excellent biocompatibility, bioactivity and chemical similarity to the inorganic component of human bone tissues. However, synthetic HA is brittle with poor mechanical toughness, thereby limiting its clinical applications. Therefore, synthetic HA finds clinical applications either as a surface coating for metallic implants or as a filler material for the polymer composites. The polymer matrix offers advantages like high flexibility, light weight and good processability [4,5]. Bonfield and coworkers developed the HAPEX™ composite consisting of 40 vol % HA microparticles dispersed in a high-density polyethylene (HDPE) matrix. This biocomposite is mainly used for orbital floor prosthesis, middle ear implant and maxillofacial surgery, because its mechanical modulus and strength are poorer than those of human cortical bones [6,7]. Generally, the mechanical performance of HA/polymer composites can be improved by reinforcing with hydroxyapatite nanoparticles rather than HA microparticles. In recent years, ceramic nanomaterials with enhanced biological, mechanical and physical properties can be synthesized because of the advances in nanotechnology. Such nanoparticles promote osteoblastic adhesion and proliferation due to enhanced cell protein-material interactions [8,9]. In particular, hydroxyapatite nanoparticles with good biocompatibility have been added to non-degradable and degradable polymers to form biocomposites [10,11,12].

Since the successful exfoliation of the graphene layer from graphite by Novoselov et al. using a simple scotch tape technique [13], the properties and applications of graphene have received enormous attention recently. Although this technique can produce high purity graphene, however, low production yield limits its application as a filler material for polymers. The low cost and massive scalability of graphene can be prepared using chemical oxidation of graphite flakes in strong acids to give graphene oxide (GO) [14], followed by either chemical or thermal reduction treatment to generate reduced GO. The basal plane carbon atoms of GO bind with epoxide and hydroxyl groups, while its edge carbon atoms with carboxyl and carbonyl groups [15]. Those functional groups can enhance interfacial bonding between the GO and polymeric matrix, leading to efficient stress transfer across the polymer-GO interface during mechanical tests. Consequently, the two-dimensional graphene-based material with a high mechanical modulus and strength is an ideal nanofiller for reinforcing biopolymers [16,17]. Furthermore, GO-reinforced polymers have also been found to exhibit good biocompatibility [17,18,19,20]. Pinto et al. reported that a small amount GO addition to polylactic acid (PLA) enhances the adhesion and proliferation of fibroblast on GO/PLA film [20]. This is because GO with hydroxyl and carboxyl groups increases the hydrophilicity of the PLA film, thereby facilitating cell-material interactions. Enhanced hydrophilicity promotes the adhesion of some proteins, like vitronectin and fibronectin. Fibronectin in the ECM is involved in the binding with cell surface integrins and induces the reorganization of the actin cytoskeleton, which is essential for cell proliferation.

Electrospinning is an economical, simple and versatile technique to deposit polymer fibers with dimensions from micrometers down to nanometers onto a target using an electric field to regulate the ejection of the polymeric fluid jet from the syringe [21,22,23,24]. Electrospun scaffolds with a nanofibrous feature having interconnecting pores and a large surface to volume ratio show morphological similarities to the natural ECM [3,25,26]. The electrospun mats with large surface areas favor cell attachment, so the need for a second surgery to remove the scaffolds is eliminated. Electrospun nanofibers can be fabricated from natural and synthetic polymers. To mimic bone tissues, nanohydroxyapatite particles are added to these polymers to form nanofibrous scaffolds [26,27,28,29,30,31]. In addition, electrospun GO-polymer nanofibrous scaffolds have also been prepared very recently [17,32,33,34,35]. Furthermore, GO is very effective for enhancing the mechanical properties of biodegradable polymers. In this respect, GO and nanohydroxyapatite have been incorporated into natural polysaccharide-based polymers, such as chitosan and alginate [36,37]. Natural polymers generally suffer from low mechanical strength especially in the presence of water and humid environments. Comparing to starch-based polymers, synthetic polylactic acid (PLA) exhibits better mechanical properties. This polymer degrades through hydrolysis under the de-esterification mechanism [38,39,40,41]. Thus, it is a promising biomaterial for tissue engineering and regenerative medicine [42,43,44]. Recently, Ma et al. carried out a preliminary study on the structure and short-term 3-(4,5-dimethylthiazol-2-yl)-2,5-diphenyltetrazolium bromide (MTT) tests of electrospun PLA nanofibers reinforced with GO and hydroxyapatite nanoparticles [45]. Their MTT results indicated that both GO and hydroxyapatite nanoparticles enhance murine MC3T3-E1 cell proliferation for a 24 h test. However, the cell proliferation of their hybrid scaffolds is poorer than that of PLA after 48 h. The aims of our work are to prepare electrospun PLA-nanohydroxyapatite rod (nHA)-GO nanofibrous mats and to study their mechanical and thermal properties, as well as long-term biocompatibility.

## 2. Materials and Methods

### 2.1. Materials

PLA was purchased from Shenzhen Bright China Inc. (Shenzhen, China). Nanohydroxyapatite rod (nHA) powders were obtained commercially from Nanjing Emperor Nano Materials (Nanjing, China). Graphite flakes were bought from Sigma-Aldrich Inc. (Saint Louis, MO, USA). All reagents such as *N*,*N*-dimethylformamide (DMF), dichloromethane (DCM), K_2_MnO_4_, NaNO_3_, etc., were used as received.

### 2.2. Preparation of Graphene Oxide (GO)

Graphene oxide (GO) was prepared from the chemical oxidation of graphite flakes following a modified Hummers process. Briefly, graphite flakes were firstly added into concentrated H_2_SO_4_ with NaNO_3_ and stirred in an ice bath for 2 h. Subsequently, K_2_MnO_4_ was added to the mixed solution slowly. The reaction was stirred for 2 days. After that, H_2_O_2_/H_2_O (2.5:100 mL) was added and cooled in the ice bath. The product was centrifuged and the supernatant was decanted away. The remaining solid material was then washed with 3% HCl and water three times, respectively, followed by freeze drying.

### 2.3. Electrospun Nanofibrous Mats

**Figure 1:**
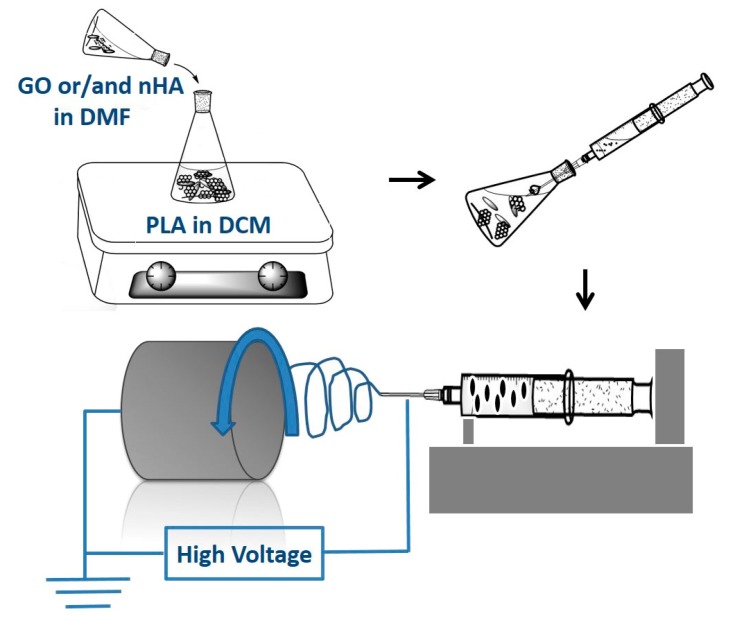
Schematic illustration showing the preparation of electrospun nanocomposite fibrous mats. PLA, polylactic acid; GO, Graphene Oxide; nHA, nanohydroxyapatite rod; DCM, dichloromethane; DMF, *N*,*N*-dimethylformamide.

To prepare pure PLA nanofibers, PLA pellets were dissolved in a 75:25 (*v*/*v*) mixture of DCM/DMF. The solution was homogenized by stirring overnight at room temperature. For preparing PLA/15 wt % HA and PLA/15 wt % HA-*x*GO (*x* = 1–3 wt %) nanofibers, HA and GO powders were weighed, dispersed in DMF under sonication for 60 min, respectively, and then mixed with PLA/DCM solution. The nHA content of 15 wt % was used in order to promote the attachment and growth of osteoblasts. Pure PLA and composite nanofibers were produced from a nanofiber electrospinning unit (NEU; Kato Tech Co., Kyoto, Japan). The polymer or composite solution was loaded into a syringe pump and connected to a stainless steel needle tip with an orifice diameter of 0.9 mm. A high voltage of 18–20 kV was applied to the needle, and the distance from the needle tip to the target collector was maintained at 12 cm. The solution was ejected at a rate of 1 mL/h in which the fibers were collected by a grounded rotating drum at 2 m/min. Figure 1 is a schematic diagram illustrating the step procedures for fabricating electrospun fibrous mats. The resulting fibrous mat was dried overnight in a vacuum dryer at 60 °C to remove solvent residue.

### 2.4. Material Characterization

The morphology of nHA was observed in a transmission electron microscope (TEM; Philips FEG CM 20, Philips, Amsterdam, The Netherlands). The final GO product was characterized using an atomic force microscope (AFM; Veeco Nanoscope V, Veeco Instruments Inc., Plainview, NY, USA) and a Raman spectrometer (LabRam, JY/Horiba, Edison, NJ, USA). The morphology of electrospun fiber mats was examined in a scanning electron microscope (SEM; Jeol JSM-820, Jeol industries, Tokyo, Japan) and TEM. The diameter and porosity of the fibers were analyzed from the SEM images by image analysis using ImageJ software (ImageJ, Bethesda, MD, USA). Porosity was evaluated by means of segmenting grey scale images under auto-threshold mode to recognize the top layer of the fiber. Fourteen measurements were made for each sample for its porosity evaluation. Fourier transform infrared (FTIR) spectra of nanofibers were collected with a Perkin Elmer spectrometer (16 PC, Perkin-Elmer Corp., Boston, MA, USA) in the wavenumber range of 400–2000 cm^−1^ with a resolution of 4 cm^−1^.

Differential scanning calorimetry (DSC, TA Instruments, New Castle, DE, USA) measurements were conducted with a TA Instruments Model 2910 under a protective nitrogen atmosphere. The specimens were first heated to 200 °C, maintained at this temperature for 3 min and cooled to 30 °C at a rate of 10 °C/min. A second heating scan to 200 °C at the same rate was subsequently initiated.

Tensile properties of electrospun fiber mats were measured with an Instron tester (Model 5567, Instron Corp., Norwood, MA, USA) using a load cell of 50 N at a crosshead speed of 10 mm/min at room temperature. All fibrous mats were cut into standard rectangular specimens of 50 mm in length and 10 mm in width [46,47]. Stress-strain curves of fibrous mats were recorded. Five samples of each composition were tested, and the average value was reported.

### 2.5. Water Uptake

PLA and its nanocomposite fibrous mats were cut into specimens of 20 mm in length and 10 mm in width and weighed before immersing in a simulated body fluid (SBF). This solution was prepared by dissolving certain amounts of chemical reagents, including Na^+^ (142 mM) , K^+^ (5 mM), Ca^2+^ (2.5 mM), Mg^2+^ (1.5 mM), Cl^−^ (147.8 mM), HCO_3_^−^ (4.2 mM), HPO_4_^−^ (1 mM) and SO_4_^2−^ (0.5 mM). It was buffered to a pH of 7.4 using tris-(hydroxymethyl)-aminomethane and 1 M HCl [48]. The fibers were rinsed with deionized water, wiped gently with filter paper and then weighed before immersion in the solution. After immersion in SBF at room temperature (23 °C) for 1, 3 and 14 days, the specimens were removed from the solution, rinsed with deionized water and weighed. The water uptake was calculated using the following equation:

(1) $$Water\;uptake\;\left(\%\right)=100\times \frac{W_{wet}-W_{0}}{W_{0}}$$

where *W*_wet_ is the weight of the wet fiber mat and *W*_0_ is the initial weight of the sample prior to immersion. Six samples of each composition were tested, and their mean standard deviation (±SD) was determined.

### 2.6. Cell Cultivation and Viability

Human osteoblast cell line Saos-2 was cultured in Dulbecco’s Modified Eagle Medium (DMEM; Thermo Scientific, Pittsburgh, PA, USA) with 10% fetal bovine serum, 100 mg/mL of streptomycin and 100 U/mL of penicillin. The fibrous mats were sliced into round disks of 6 mm in diameter and sterilized with 70% ethanol before cell cultivation. By rinsing three times with sterile phosphate-buffered saline (PBS) solution, the samples were dipped in DMEM medium overnight and then placed into the 96-well plates in triplicate followed by seeding with 100 μL cell suspension containing 1 × 10^4^ cells per well. These plates were placed in an incubator at 37 °C with humidified atmosphere of 95% air and 5% CO_2_ for 5 and 7 days, respectively. At the end of each time point, samples were taken out from the wells and rinsed with PBS solution twice to remove the unattached cells, fixed with 10% formaldehyde solution and dehydrated in a series of ethanol solutions (30, 50, 70, 90, 100 vol %) followed with air drying. Finally, samples were coated with a thin gold film for SEM observation.

The cell viability of PLA, PLA/15%nHA and PLA/15%nHA-GO fibrous mats was assessed with 3-(4,5-dimethylthiazol-2-yl)-2,5-diphenyltetrazolium bromide (MTT) colorimetric assay. After culturing the samples with the 100 μL cell suspension at 1 × 10^4^ cells/well, the plates were incubated at 37 °C in a humidified atmosphere of 95% air/5% CO_2_ for 3, 7 and 10 days. The DMEM medium was changed every three days. After each incubation time, the medium was aspirated, and 100 μL of MTT solution were added into each well to give insoluble formazan crystals. Prior to adding 100 μL of 10% sodium dodecyl sulfate (SDS) in 0.01 M hydrochloric acid to dissolve the crystals, the plates were incubated for another 4 h. The optical absorbance of the solubilized formazan was analyzed with a multimode detector (Beckman Coulter DTX 880, Beckman Coulter Inc., Fullerton, CA, USA) at a wavelength of 570 nm with a reference wavelength of 640 nm. The mean standard deviation (±SD) of five replicates was determined. A two-way analysis of variance (ANOVA) was used to evaluate the statistical data; a *p*-value of 0.05 was selected as the level of significance.

### 2.7. Alkaline Phosphatase

Alkaline phosphatase is an enzyme secreted by osteoblasts acting as the marker to reveal earlier osteoblastic differentiation for bone tissue mineralization. Samples were sliced into disks of 14 mm in diameter, sterilized with ethanol, rinsed with PBS and placed in the 24-well plate. Then, 10^4^ cells/well were introduced to the culture plate followed by incubation at 37 °C in an atmosphere of 95% air/5% CO_2_ for 3, 7 and 14 days. The culture medium was refreshed every three days. At the end of each incubation period, the cells were washed with PBS three times and lysed with 0.1% Triton X-100 at 4 °C for 15 min. The cell lysates were then transferred to 1.5 mL tubes and centrifuged at 4 °C for 10 min. Subsequently, 10 μL of the supernatant of each sample were transferred to a 96-well plate. The alkaline phosphatase (ALP) activity was determined with a commercial assay kit (No. 2900, Stanbio Laboratory, Boerne, TX, USA) employing colorless *p*-nitrophenyl phosphate (pNPP) as a phosphatase substrate. In the process, ALP enzyme hydrolyzed the substrate to yellow *p*-nitrophenol and phosphate. The absorbance was recorded with a multimode detector at a wavelength of 405 nm. The ALP activity was normalized to the protein level of each sample lysate measured by the Bio-Rad Protein Assay (Bio-Rad, Hercules, CA, USA). A two-way ANOVA was used to analyze the statistical data; a *p*-value of 0.05 was selected as the level of significance.

## 3. Results and Discussion

### 3.1. Nanomaterial and Electrospun Fiber Features

**Figure 2:**
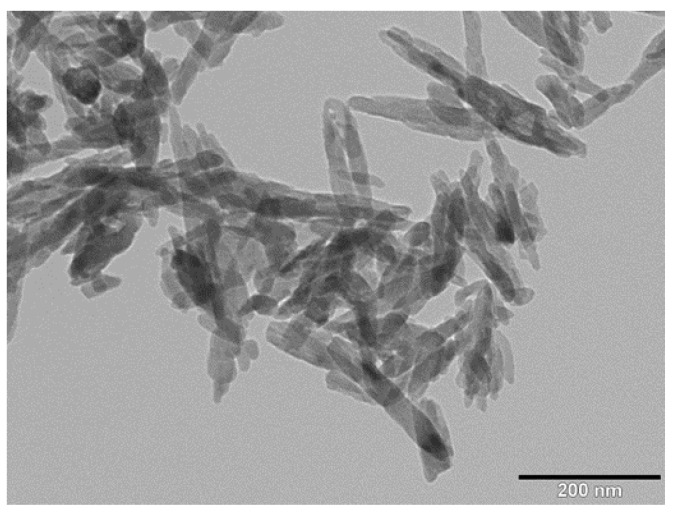
TEM image of nanohydroxyapatite rod (nHA).

**Figure 3:**
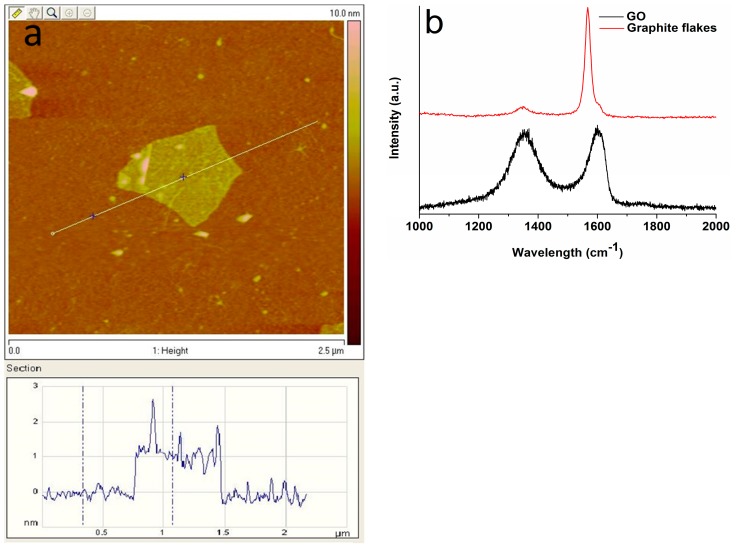
(**a**) AFM image of GO with height profile across a scan line and (**b**) Raman spectra of GO and graphite.

The transmission electron microscopy image revealed nHA exhibiting a width of 20 nm and a length of 100 nm (Figure 2). Figure 3a shows the AFM image of GO deposited as a monolayer sheet onto a silicon substrate, along with its height profile. The thickness of the GO sheet is 1 nm, but some regions display a height profile over 1 nm due to the oxygenated groups of GO. The Raman spectrum of GO shows the presence of the D band (1340 cm^−1^) and the G band (1595 cm^−1^) (Figure 3b). The D band is related to the presence of the defect created by the oxygenated functional groups on the carbon basal plane, and the G band is due to the ordered sp^2^-bonded carbon atoms [49]. By contrast, graphite flake exhibits a sharp G band at 1580 cm^−1^ and a weak D band (1340 cm^−1^) due to disorders associated with strong C–C bonding and impurity [50]. Furthermore, the G band in GO is shifted to a higher wave number (1595 cm^−1^) due to the oxygenation of graphite [51].

**Figure 4:**
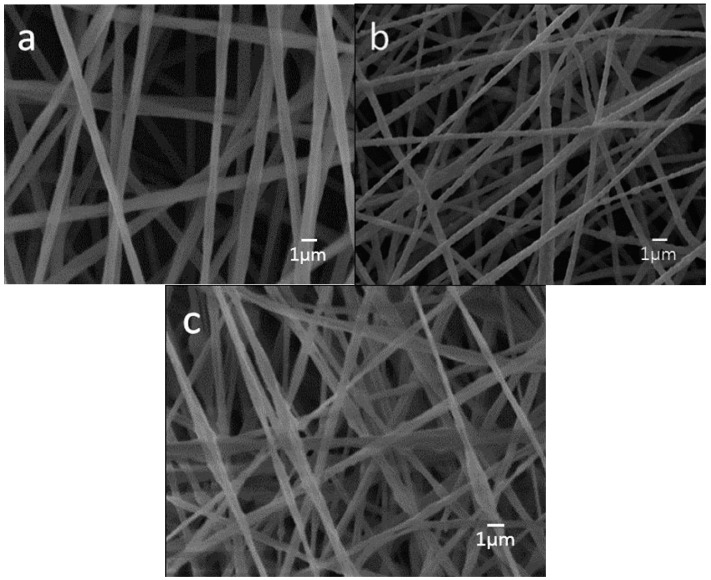
SEM micrographs of electrospun (**a**) PLA; (**b**) PLA/15%nHA and (**c**) PLA/15%nHA-3%GO fibrous mats.

Figure 4a–c is the representative SEM micrographs showing the morphologies of neat PLA, PLA/15%nHA and PLA/15%nHA-3%GO fibrous mats. PLA displays a relatively smooth feature having an average fiber diameter of 786 ± 189 nm, as determined by ImageJ software (Figure S1a). The surface of the PLA/15%nHA nanocomposite mat is somewhat rougher than pure PLA. The mean diameter of PLA/15%nHA nanocomposite fibers is 563 ± 196 nm (Figure S1b). The addition of 15% nHA to PLA reduces the diameter of fibers slightly. This is due to the reduction of polymer concentration and the change of solution viscosity by adding 15% nHA [52]. By incorporating 3% GO to the PLA/15%nHA, the diameter of the composite fibers further decreases to 412 ± 240 nm (Figure S1c). An agglomeration of 15% nHA and GO at the needle tip would reduce the effective orifice diameter of the needle, thereby producing nanofibers with a finer diameter [53]. Furthermore, porosity is another key factor for ideal scaffolds for bone regeneration. The results of porosity and fiber diameter of all fibrous mats as determined by ImageJ software are tabulated in Table 1.

**Figure 5:**
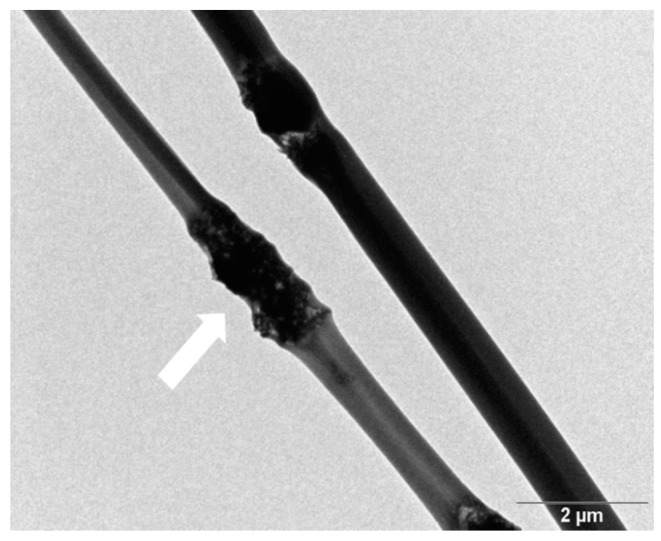
TEM micrograph of the PLA/15%nHA-3%GO nanocomposite fibrous mat. Fillers of PLA/15%nHA-3%GO fiber are indicated by an arrow.

Polymer nanocomposites are generally reinforced with low nanofiller contents, say 1–3 wt % for achieving the desired functional properties [54,55,56,57]. In the case of biocomposites for bone replacement and regeneration applications, higher nHA content, i.e., 15–18 wt %, is needed to promote the adhesion and growth of osteoblasts on the composite surfaces [11,12]. Such large nHA loading inevitably would induce the aggregation of fillers and the formation of beads in electrospun fibers. Figure 5 is the TEM image showing the morphology of PLA/15%nHA-3%GO nanocomposite fibers. It is apparent that the fillers form aggregates inside PLA/15%nHA-3%GO fibers as indicated by an arrow.

**Figure 6:**
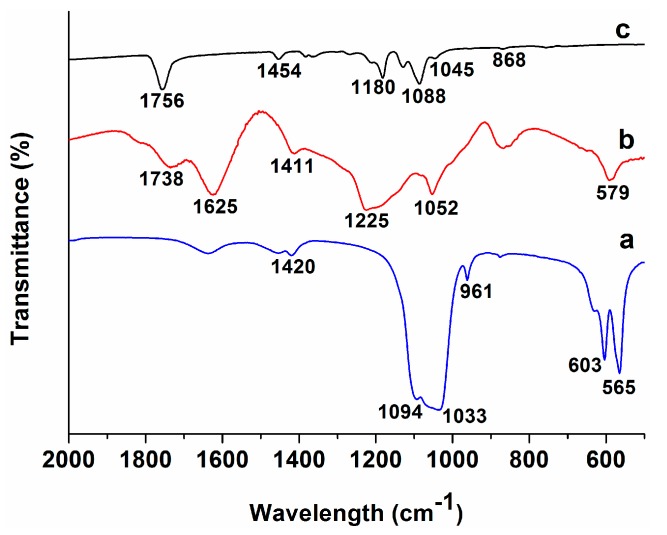
FTIR spectra of (**a**) nHA; (**b**) GO and (**c**) pure PLA specimens.

**Figure 7:**
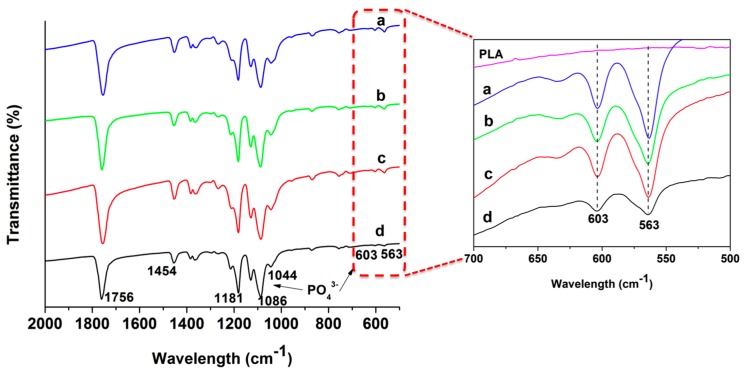
FTIR spectra of (**a**) PLA/15%nHA; (**b**) PLA/15%nHA-1%GO; (**c**) PLA/15%nHA-2%GO and (**d**) PLA/15%nHA-3%GO nanocomposite fibers. The enlarged spectra in the wave number ranging from 500 to 700 cm^−1^ are presented.

Figure 6 shows the FTIR spectra of pure PLA, GO and nHA specimens. The spectra of PLA/15%nHA and PLA/15%nHA-*x*%GO nanocomposite mats with 1%–3% GO are shown in Figure 7. Pure PLA shows a main C=O vibration peak at 1756 cm^−1^, CH_3_ asymmetrical scissoring at 1454 cm^−1^, C–O asymmetrical stretching and CH_3_ twisting at 1180 cm^−1^, C–O–C stretching at 1088 cm^−1^, C–CH_3_ stretching at 1045 cm^−1^ and C–COO stretching at 868 cm^−1^ [58,59]. For the nHA specimen, the peak at 961 cm^−1^ is caused by the γ1-mode vibration; the 1094 and 1033 cm^−1^ bands relate the γ_3_-mode of P–O symmetric stretching vibration; and the 565 and 603 cm^−1^ bands correspond to the γ_4_ P–O bending vibration [60]. The band at 1420 cm^−1^ is attributed to the CO_3_^2^^−^ group due to the absorption of carbon dioxide from the atmosphere into solution during the nHA synthesis [61]. From these, ν_1_ relates to the nondegenerate stretching PO, and ν_3_ and ν_4_ refer to the triply degenerate stretching [62]. The oxygenated groups of GO induce C=O carbonyl stretching at 1734 cm^−1^, C=C stretching at 1625 cm^−1^, C–OH stretching at 1411 cm^−1^, C–O–C vibration at 1225 cm^−1^ and C–O stretching at 1052 cm^−1^ [63]. These functional groups render GO highly hydrophilic. For electrospun PLA/15%nHA and PLA/15%nHA-*x*%GO fibers, several absorption bands at 563, 603 and 1094 cm^−1^ due to the PO_4_^3−^ group of nHA can be observed. The FTIR spectra in the range of 500–700 cm^−1^ clearly reveal the presence of 563 and 603 cm^−1^ bands in these fibers.

Thus FTIR characterization confirms the presence of HA in electrospun PLA/15%nHA and PLA/15%nHA-*x*%GO nanocomposite fibers. For these composite fibers, the inclusions of nHA and GO into PLA give rise to the presence of characteristic bands of individual material components, but with a slight shift in the peak values due to the overlap of some bands. The 1044 cm^−1^ peak in the nanocomposite fiber is ascribed with the overlap of C–CH_3_ stretching of PLA at 1045 cm^−1^, the γ_3_-mode of P–O group of nHA at 1033 cm^−1^ and the C–O stretching of GO at 1052 cm^−1^. Moreover, the 579 cm^−1^ of GO overlaps with the 563 cm^−1^ peak of nHA, and the 1738 cm^−1^ band of GO overlaps with the 1756 cm^−1^ peak of PLA. The small amount of GO additions and the overlapping of its characteristic bands with nHA and PLA make it difficult to identify GO bands of nanocomposite scaffolds.

### 3.2. Thermal Behavior

**Figure 8:**
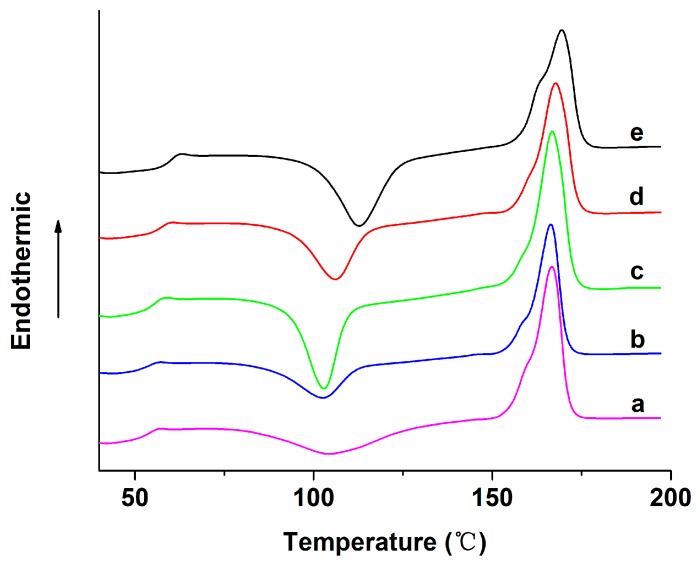
Second heating curves of electrospun (**a**) PLA; (**b**) PLA/15%nHA; (**c**) PLA/15%nHA-1%GO; (**d**) PLA/15%nHA-2%GO and (**e**) PLA/15%nHA-3%GO fibrous mats.

Figure 8 shows the DSC heating traces of pure PLA, PLA/15%nHA and PLA/15%nHA-*x*%GO fibrous mats. The glass transition temperature (*T*_g_), cold crystallization temperature (*T*_cc_), melting temperature (*T*_m_), cold crystallization enthalpy (Δ*H*_cc_) and melting enthalpy (Δ*H*_m_) can be obtained from secondary heating traces and are tabulated in Table 2. The degree of crystallinity (*Χ*_c_) of PLA and its nanocomposites is evaluated from the following equation,

(2) $$X_{c}\;\left(\%\right)=\left(\frac{\Delta H_{m}-\Delta H_{cc}}{\Delta H_{m}^{o}\left(1-\Phi \right)}\right)\cdot 100$$

where $\Delta H_{m}^{o}$ is the melting enthalpy of totally crystallized (100%) PLA, i.e., 93 J/g [64], and Φ is the weight fraction of the composite filler. The *Χ*_c_ values of the specimens investigated are also summarized in Table 2.

Electrospun PLA fibers experience slow crystallization because of rapid solvent vaporization and fast cooling during spinning. Upon the second heating of PLA mat during the measurement, an exothermic peak (104.4 °C) appears in the heating scan due to a cold crystallization process associated with the rearrangement of amorphous molecules into a crystalline phase. Cold crystallization occurs above the glass transition temperature, but well below the melting temperature of PLA. Mezghani and Spruiell reported that amorphous polymers generally have a higher tendency to undergo this transition compared to semi-crystalline polymers. Thus, amorphous polymers have a large Δ*H*_cc_, while semi-crystalline polymers exhibit small Δ*H*_cc_ [65]. In this study, the Δ*H*_cc_ of PLA is large and comparable to that reported in the literature [66,67]. The incorporation of 15 wt % nHA into pure PLA decreases the *T*_cc_ value from 104.4 to 102.7 °C. A decrease in the *T*_cc_ value in the heating scan implies that nHA facilitates the crystallization process of PLA at a lower temperature. Thus, nHA acts as the site for nucleating PLA molecules. Further addition of 1% GO to PLA/15%nHA slightly increases *T*_cc_ to 102.9 °C. A large increment in the *T*_cc_ value, i.e., 112.9 °C, is found in the PLA/15%nHA-3%GO fibrous mat, indicating that the filler with higher GO content containing the oxygenated group retards PLA crystallization. Another reason is due to the agglomeration of fillers in the fibrous mat containing high filler content, as mentioned above.

From Table 2, it can be seen that the *T*_g_ of PLA increases as a result of GO and/or nHA additions. Moreover, the degree of crystallinity of PLA increases as a result of 15% nHA addition, demonstrating the effective nucleation effect of nHA. However, the *X*_c_ value decreases considerably by incorporating 1%–3% GO into PLA/15%nHA. In particular, the PLA/15%nHA-3%GO fibrous mat exhibits lowest the *X*_c_ value of 4.32%. The relatively high filler content and associated filler aggregation in this nanocomposite fibers leads to the immobilization of PLA molecules, thus causing physical hindrance for crystallization and giving rise to the lowest crystallinity.

### 3.3. Tensile Behavior

**Figure 9:**
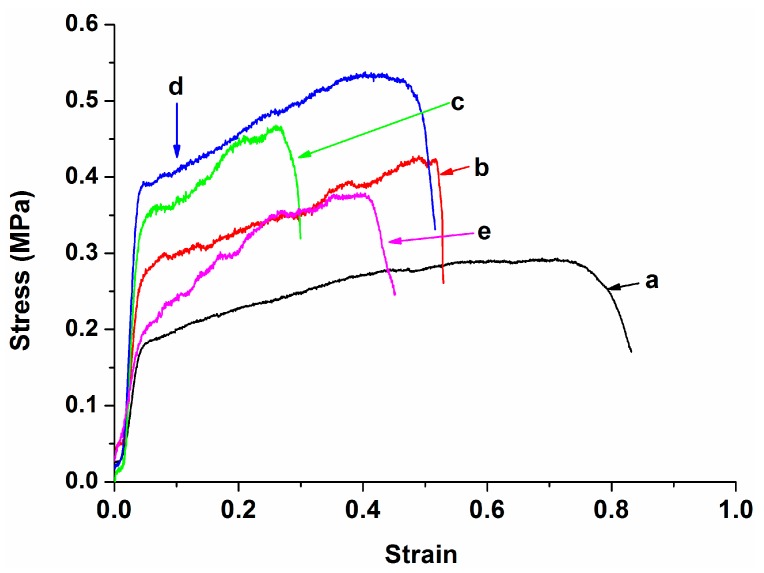
Tensile stress–strain curves of electrospun (**a**) PLA; (**b**) PLA/15%nHA; (**c**) PLA/15%nHA-1%GO; (**d**) PLA/15%nHA-2%GO and (**e**) PLA/15%nHA-3%GO fibrous mats.

Figure 9 shows the stress-strain curves of PLA, PLA/15%nHA and PLA/15%nHA-*x*%GO fibrous mats. The tensile strength and Young’s modulus of these specimens are listed in Table 3. Apparently, the tensile strength and elastic modulus of the electrospun PLA fibrous mat are improved by adding 15% nHA. This demonstrates that the nHA fillers can bear the applied load because of an effective stress-transfer mechanism. By adding 1% GO to the PLA/15%nHA, a dramatic improvement in the modulus is observed, i.e., 28.4% increment. Further modulus enhancement to 69.3% can be achieved by incorporating 2% GO into the PLA/15%nHA. This implies that GO sheets with a high elastic modulus and a large surface area can stiffen and reinforce the PLA fibrous mat effectively. At 3% GO loading, the stiffness and tensile strength of the PLA/15%nHA-3%GO fibrous mat drop sharply due to the filler agglomeration.

It is generally known that the graphene monolayer exhibits an exceptionally high elastic modulus of 1 TPa and a tensile strength of 130 GPa [68]. However, the presence of oxygenated groups in GO reduces the modulus to 380–470 GPa or even lower and the tensile strength to 87.9 MPa [69,70,71]. The modulus and strength of GO are much higher than those of pure PLA, i.e., modulus of 3.5 GPa and tensile strength of 48 MPa [72]. Thus GO acts as an effective reinforcement for the PLA. Apparently, the presence of porosity in electrospun PLA reduces the modulus from 3.5 GPa to 8.58 MPa and the tensile strength from 48 to 0.27 MPa. The tensile modulus and strength of the electrospun PLA fibrous mat in this study agree reasonably well with those of the electrospun PLA mat reported by Dong et al. [67]. From the literature, GO reinforces polymers, such as polycaprolactone (PCL), polyvinyl alcohol and polyamide-12, effectively at low loading levels [73,74]. In general, scaffolds for bone tissue engineering applications should possess high mechanical strength and stiffness, so that they can provide load support for osteoblastic adhesion and proliferation during bone tissue regeneration. From Table 3, the hybridization of 15% nHA with 2% GO enhances both the stiffness and strength of the PLA fibrous mat, rendering the PLA/15%nHA-2%GO fibrous mat a promising material for bone scaffold applications.

### 3.4. Water Absorption

**Figure 10:**
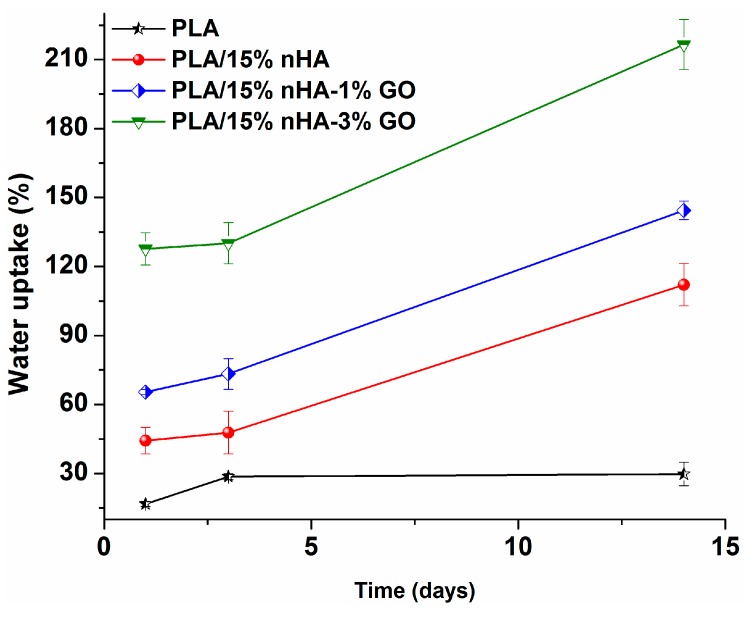
Water absorption behavior of PLA and PLA-based composite nanofiber mats.

Figure 10 shows the variation of water uptake with immersion time for the PLA and its nanocomposite fibrous mats. The pure PLA mat displays low water uptake due to the presence of the methyl group in its structure. In other words, the methyl group renders PLA exhibiting hydrophobic behavior. Water uptake is initiated in the amorphous regions of PLA, since it is less organized and more accessible to water molecules [75]. PLA composites show large water intake due to their GO and/or nHA fillers, particularly those with GO additions. Therefore, the surface wettability of electrospun composite fiber mats is enhanced by the GO and/or nHA inclusions, which in turn assists cell seeding and proliferation as a result of enhanced protein adsorption [76,77,78].

### 3.5. Cell Cultivation and Proliferation

**Figure 11:**
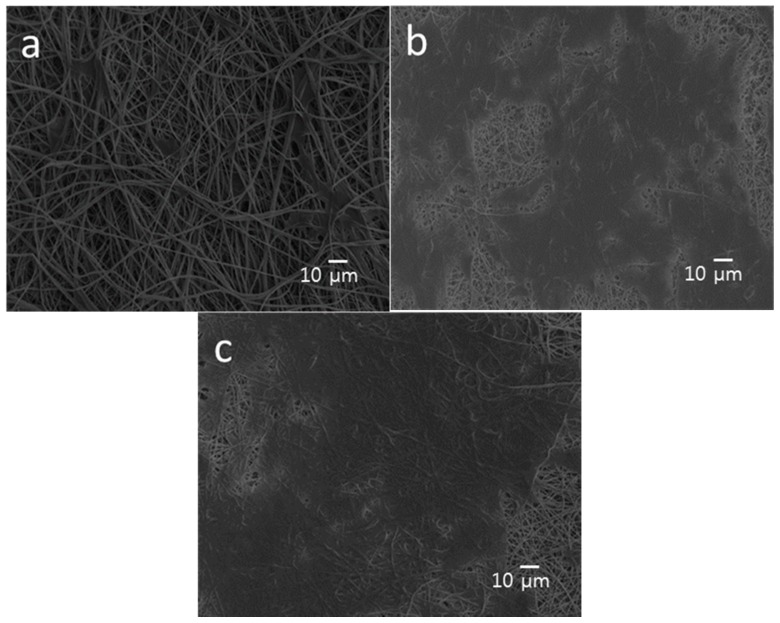
SEM images showing the attachment of osteoblasts on (**a**) PLA; (**b**) PLA/15%nHA and (**c**) PLA/15%nHA-2%GO fibrous mats.

Figure 11a–c show the SEM micrographs of PLA, PLA/15%nHA and PLA/15%nHA-2%GO fibrous mats cultivated with osteoblasts for five days. For neat the PLA mat, a few osteoblasts attach on its surface as expected. This is because the neat PLA mat lacks the sites for cell adhesion. However, many cells anchor, grow and spread flatly on the PLA/15%nHA mat, so several neighbor cells link to each other through cytoplasmic extension. The nHA fillers of nanoscale dimension provide effective seeding sites for osteoblasts. As mentioned above, GO and/or 15% nHA fillers change the hydrophobic PLA mat to a moderate hydrophilic behavior by enhancing water absorption. Surfaces with moderate hydrophilicity facilitate the adsorption of proteins released from osteoblasts, while hydrophobic surfaces show poor cell attachment [76].

**Figure 12:**
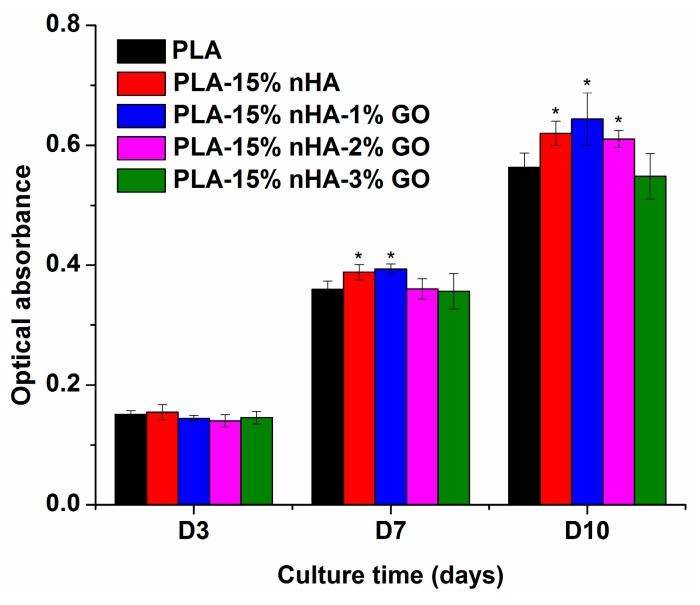
The MTT assay results of Saos-2 cells cultured on neat PLA and its composite fibrous mats for 3, 7 and 10 days. * *p* < 0.05.

Figure 12 shows the MTT results revealing the proliferation of osteoblasts on the PLA, PLA/15%nHA and PLA/15%nHA-*x*%GO fibrous mats. The optical absorbance is related to cell proliferation on the specimen surfaces [79]. At Days 7 and 10, this figure shows that the PLA/15%nHA, PLA/15%nHA-1%GO and PLA/15%nHA-2%GO mats exhibit higher cell proliferation when compared to neat PLA. Among these, the PLA/15%nHA-1%GO fibrous mat displays the highest cell proliferation. These results demonstrate that the nHA and GO nanofillers exert a significant effect on the adhesion and proliferation of osteoblastic cells on nanocomposite fibrous mats. Moreover, hybridization of 15% nHA with 1% GO gives the best results. As mentioned above, Ma et al. carried out MTT murine bone cell tests for PLA and hybrids with GO and hydroxyapatite particles for a short term of one and two days. Their results showed that GO and hydroxyapatite fillers are beneficial for cell proliferation on PLA for one day only. At Day 2, their hybrids show poorer biocompatibility than PLA. Thus, long-term cell proliferation is needed to evaluate the cell viability of the scaffolds for clinical use, as shown in Figure 12.

### 3.6. Alkaline Phosphatase

**Figure 13:**
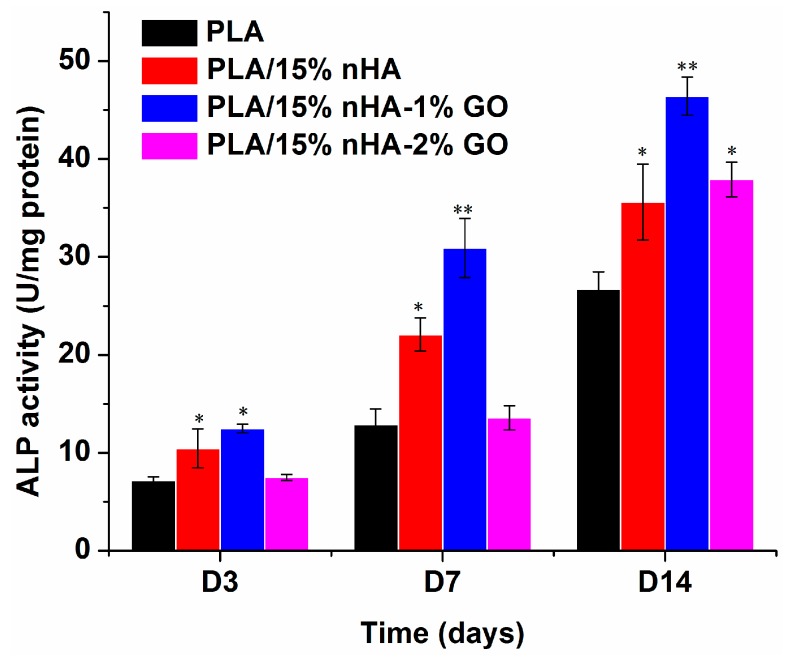
ALP activity of Saos-2 cells cultured on neat PLA and its composite fibrous mats for 3, 7 and 14 days. * *p* < 0.05, ** *p* < 0.01.

Figure 13 shows the ALP activity for neat PLA, PLA/15%nHA, PLA/15%nHA-1%GO and PLA/15%nHA-2%GO fibrous mats. At Days 7 and 14, the results indicate the good ALP activity level of osteoblasts on the fibrous PLA/15%nHA mat compared to neat PLA. This is attributed to the nHA fillers promoting the adhesion and proliferation of osteoblasts greatly. Furthermore, synthetic nHA exhibits excellent osteoconductivity and biocompatibility. On the basis of the proliferation/differentiation model reported by Stein and Lian, cells largely grow up to 7–14 days and then begin to secrete ECM proteins and yield early differentiation ALP markers [80]. Figure 11 also reveals that the ALP activity of the PLA/15%nHA mat increases considerably by adding 1% GO. As shown in Figure 13, hybridization of 15% nHA with 1% GO gives the highest osteoblastic proliferation or no cell toxicity for the composite fibrous mat. As a result, the combination of nHA with GO can give PLA with the highest cell compatibility. From the literature, graphene-based materials have been found to promote bone marrow-derived mesenchymal stem cells’ attachment and differentiation via protein-material interactions [81,82]. Furthermore, GO acts synergistically with calcium phosphate, thereby increasing ALP activity and the calcium deposition of osteoblasts [83].

## 4. Conclusions

In this article, we investigated the preparation, morphological, biochemical, mechanical and thermal properties of electrospun PLA scaffolds reinforced with GO and/or nHA nanofillers for bone tissue engineering applications. Image analysis revealed that the nHA and GO additions refine the diameter of electrospun PLA fibers. DSC results showed that nHA fillers facilitate the crystallization process of PLA, thus acting as the site for nucleating PLA molecules. The addition of 15% nHA to PLA substantially increased its tensile strength and elastic modulus. Furthermore, the nanocomposite scaffold with 15% nHA and 1% GO hybrid fillers exhibited good tensile performance. The nHA and GO nanofillers enhanced the water uptake of PLA. Cell cultivation, MTT and ALP tests demonstrated that the nanocomposite scaffolds exhibit higher cell proliferation than the pure PLA mat, particularly for the scaffold with 15% nHA and 1% GO nanofillers. On the basis of these results, the novel electrospun PLA nanocomposite scaffold reinforced with 15% nHA and 1% GO with the high tensile strength and modulus, as well as excellent cell proliferation, is an attractive biomaterial for bone tissue engineering applications.

## Figures and Tables

**Table 1:** Average diameter and porosity of electrospun PLA and its nanocomposite fibrous mats.

Specimen	Average diameter (nm)	Porosity (%)
PLA	786 ± 189	70.52
PLA/15%nHA	563 ± 196	74.52
PLA/15%nHA-1%GO	516 ± 206	75.58
PLA/15%nHA-2%GO	502 ± 213	76.19
PLA/15%nHA-3%GO	412 ± 240	77.96

**Table 2:** Thermal parameters of the samples investigated.

Specimen	*T*_g_ (°C)	*T*_cc_ (°C)	Δ*H*_cc_ (°C)	*T*_m_ (°C)	Δ*H*_m_ (°C)	*X*_c_ (°C)
PLA	56.2	104.4	19.7	166.8	37.8	19.5
PLA/15%nHA	56.7	102.7	17.7	166.4	24.8	21.6
PLA/15%nHA-1%GO	58.1	102.9	23.9	166.7	35.3	14.6
PLA/15%nHA-2%GO	59.8	106.1	21.7	167.8	33.2	14.9
PLA/15%nHA-3%GO	62.5	112.9	27.8	169.5	31.1	4.3

**Table 3:** Tensile properties of electrospun PLA and PLA-based nanocomposite fibrous mats.

Specimen	Elastic modulus (MPa)	Tensile stress (MPa)
PLA	8.58 ± 0.53	0.27 ± 0.04
PLA/15%nHA	9.88 ± 0.31	0.41 ± 0.05
PLA/15%nHA-1%GO	12.69 ± 0.86	0.47 ± 0.03
PLA/15%nHA-2%GO	16.73 ± 0.21	0.57 ± 0.04
PLA/15%nHA-3%GO	8.10 ± 0.50	0.38 ± 0.03

## Supplementary Materials

Supplementary Materials can be found at www.mdpi.com/2073-4360/8/8/287/s1.
