# Supplementary material for: Novel Electrospun Polylactic Acid Nanocomposite Fiber Mats with Hybrid Graphene Oxide and Nanohydroxyapatite Reinforcements Having Enhanced Biocompatibility
Source: Polymers (Basel). 2016 Aug 8;8(8):287. doi: 10.3390/polym8080287 (PMC6432366; doi:10.3390/polym8080287)
Supplement: Supplementary file 1 [file polymers-08-00287-s001.pdf]

Polymers **2016**, *8*, 287; doi:10.3390/polym8080287

Supplementary Materials: Novel Electrospun Polylactic Acid Nanocomposite Fiber Mats with Hybrid Graphene Oxide and Nanohydroxyapatite Reinforcements Having Enhanced Biocompatibility

Chen Liu, Hoi Man Wong, Kelvin Wai Kwok Yeung and Sie Chin Tjong

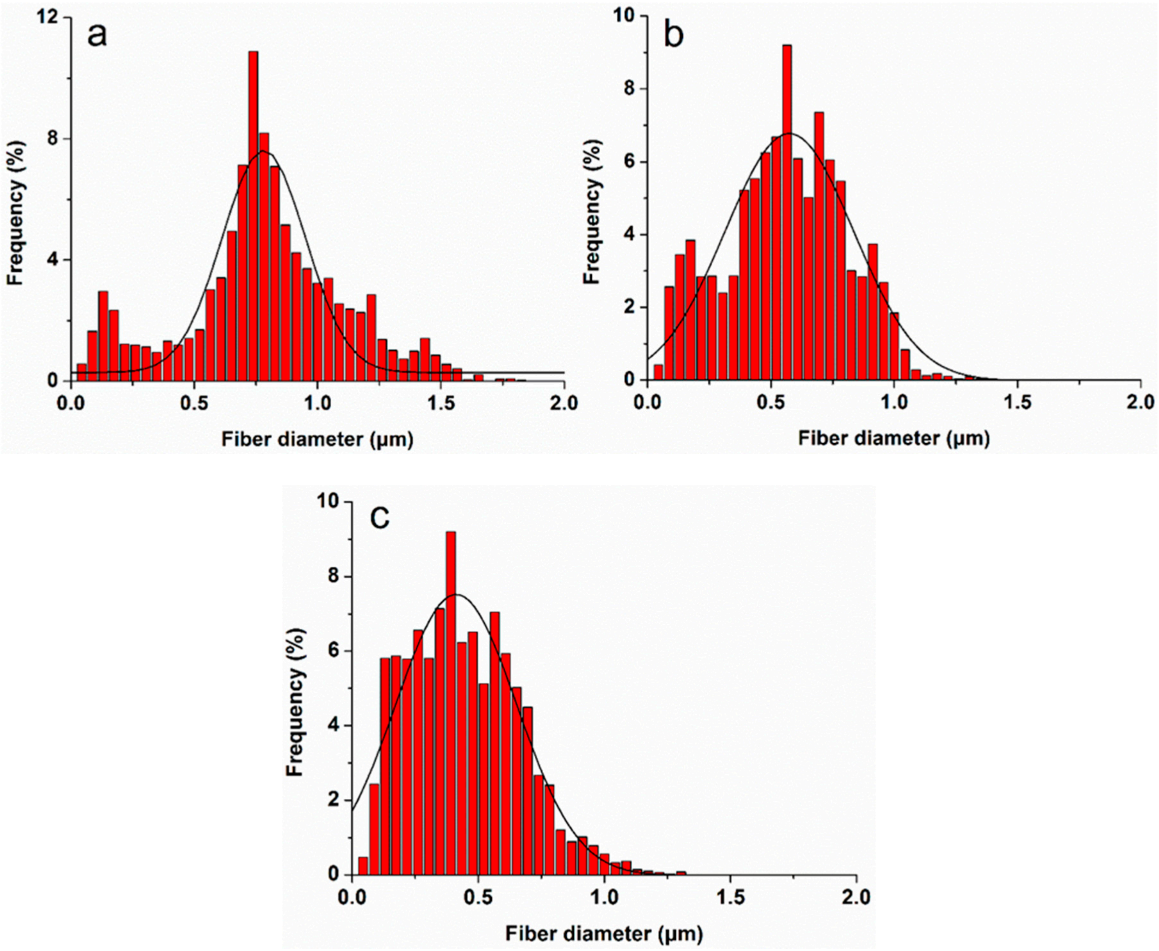

Figure S1. Fiber diameter distribution of electrospun (**a**) PLA, (**b**) PLA/15%nHA and (**c**) PLA/15%nHA-3%GO fibrous mats determined by ImageJ software.

© 2016 by the authors; licensee MDPI, Basel, Switzerland. This article is an open access article distributed under the terms and conditions of the Creative Commons Attribution (CC-BY) license (http://creativecommons.org/licenses/by/4.0/).
